# Emergence of multidrug-resistant *Staphylococcus epidermidis* in Nile tilapia (*Oreochromis* spp.): virulence, antimicrobial resistance, and nanoparticle-based control

**DOI:** 10.1186/s12917-025-05213-w

**Published:** 2026-02-02

**Authors:** Mahmoud Abou-Okada, Engy Taha

**Affiliations:** Department of Aquatic Animal Medicine and Management, Faculty of Veterinary Medicine, Cairo University, Giza, 11221 Egypt

**Keywords:** Tilapia, *Staphylococcus epidermidis*, Tilapia lake virus (TiLV), Pathogenicity, Multidrug-resistant bacteria, Silver nanoparticles (AgNPs)

## Abstract

**Background:**

*Staphylococcus epidermidis* represents an emerging zoonotic threat impacting aquatic ecosystems, livestock, and human health. This study investigated the causative agent behind summer mortality episodes affecting cultured *Oreochromis* spp. (180 ± 20 g) in Egyptian fish farms, where diseased specimens exhibiting characteristic ulcerative dermatopathy were collected from Ismailia governorate.

**Results:**

Comprehensive diagnostics excluded TiLV and NNV, while bacteriological analysis identified Gram-positive cocci producing distinctive white hemolysis-negative colonies on blood agar and red colonies on mannitol salt agar. Polyphasic characterization confirmed the isolates as *S. epidermidis* (16 S rRNA GenBank MN153038), marking the first genomic record of this pathogen in tilapia. Antimicrobial profiling revealed alarming multidrug resistance (54.5% of tested agents), including resistance to β-lactams (oxacillin, ampicillin, and cefoxitin) that suggests possible methicillin-resistant (MRSE) phenotypes, despite retained vancomycin susceptibility (MIC = 4 µg/mL). Controlled challenge trials demonstrated dose- and route-dependent virulence, with scale removal during immersion exposure precipitated 95% mortality in fingerlings (2.3 ± 0.75 g) versus 55% in intact fish. In contrast, intraperitoneal injection caused 40–50% mortality in adults/juveniles. Notably, silver nanoparticles (AgNPs) exhibited size-dependent antimicrobial activity: 10-nm AgNPs showed superior efficacy (MIC = 1.25 µg/mL; MBC = 2.5 µg/mL) compared to 100-nm AgNPs (MIC = 10 µg/mL) and zinc oxide nanoparticles (MIC = 125 µg/mL). The enhanced activity of smaller AgNPs is attributed to their greater surface area and improved biofilm penetration.

**Conclusion:**

These results highlight *S. epidermidis* as an emerging threat in tilapia aquaculture, particularly given its multidrug resistance. The demonstrated efficacy of AgNPs, especially at smaller particle sizes, offers a promising alternative for controlling such resistant infections in aquaculture settings.

**Supplementary Information:**

The online version contains supplementary material available at 10.1186/s12917-025-05213-w.

## Introduction

Egypt plays a significant role in the global aquaculture industry, particularly in the farming of tilapia (*Oreochromis* spp.). As the third largest producer worldwide, the country has established itself as a key player in tilapia production, leveraging its abundant water resources and favorable climate. Furthermore, the extensive cultivation of tilapia not only contributes to the local economy but also addresses food security and nutritional needs for the growing population [1, 2]. Nevertheless, despite this success, the Egyptian tilapia sector encounters multiple sustainability challenges. On the one hand, the tilapia aquaculture experiences declining profitability and production efficiency due to disease outbreaks, seasonal climate variations, and sensitive ecosystems [3].

Coagulase Negative Staphylococci (CoNS) are commensal bacterial flora of skin, gastrointestinal and respiratory mucous membranes of humans and animals [4]. *Staphylococcus epidermidis* is a Gram-positive bacterium that is considered the major cause of infections associated with catheters, surgical wounds, peritonitis, osteomyelitis, and endophthalmitis [5].

*Staphylococcus epidermidis* has been reported previously as a fish pathogen in fresh, brackish and marine water fish in Japan, Taiwan, Greece, Iran, Turkey and Egypt. Mass fish kills caused by *S. epidermidis* have been reported in various cultured species, including yellow tail (*Seriola quinqueradiata*) and red seabream (*Chrysophrys major*) [6]. Similarly, such instances have been observed in grass carp (*Ctenopharyngodon idella*) [7] as well as tilapia (*Oreochromis* spp.) [8, 9]. Additionally, cases have been documented in rainbow trout *(Oncorhynchus mykiss)* [10] and in European seabass (*Dicentrarchus labrax*) and gilthead seabream (*Sparus aurata*) [11–13].

Staphylococci may be introduced to the aquatic environment through contaminated water or commercial fish feed. Staphylococcal infections in fish are induced by abrupt rise in water temperature or other multifactorial stressful circumstances in the aquatic environment. The disease appears in the spring and triggering mass fish kills throughout the summer [13]. *Staphylococcus epidermidis* infections in fish involve systemic disease characterized by septicemia, congestion and ulceration on the caudal peduncle, tail erosion, excessive secretion of mucous on the skin and exophthalmia [6, 11–14].

The pathogenicity of *Staphylococcus epidermidis* in farmed tilapia was confirmed through intraperitoneal injection (IP) of both bacterial suspensions and supernatants. When the bacterial dose exceeded 1.34 × 10⁷, mortality rate rose to 60%, and the bacteria were successfully re-isolated from the infected fish [8]. These findings suggest that *S. epidermidis* could lead to large-scale die-offs in tilapia under natural environments. Notably, heavily infected fish often displayed no initial external signs or abnormalities, though some cases exhibited exophthalmia (pop-eye) or skin and fin lesions [8].

The most commonly used antibiotics in aquaculture include florfenicol, oxytetracycline, erythromycin, amoxicillin, oxolinic acid, enrofloxacin, flumequine, and trimethoprim-sulfadiazine [15, 16]. Among coagulase Negative Staphylococci (CoNS), *S. epidermidis* harbored the most virulence factors and antibiotic resistance genes [17]. Specifically, it has shown resistance to penicillin, ampicillin, ampicillin-sulbactam, methicillin/oxacillin, erythromycin, gentamycin, oxytetracycline, streptomycin, tobramycin and oxacillin [12]. Moreover, *S. epidermidis* demonstrated the greatest phenotypic resistances, with resistance to nine out of the fourteen tested antibiotics, including penicillin, kanamycin, gentamycin, clindamycin, erythromycin, cefoxitin, trimethoprim-sulfamethoxazole, tobramycin, and fusidic acid [17].

In aquaculture, antimicrobials are primarily used to prevent and treat bacterial infections in fish [16]. However, when these substances are misused, they can contribute to the development of zoonotic antibiotic-resistant bacteria, posing a risk to humans through contaminated food [18]. Moreover, the improper application of antimicrobials has accelerated the spread of antimicrobial resistance (AMR), affecting not only animals but also humans and the environment [19, 20].

Nanoparticles (NPs) are increasingly recognized for their strong antimicrobial properties, which stem from multiple mechanisms of action [21, 22]. In aquaculture, various metal nanoparticles (MNPs) have been employed due to their effectiveness against bacterial pathogens [23–25]. Notably, silver nanoparticles (AgNPs) have emerged as a particularly potent antimicrobial agent, demonstrating broad-spectrum bactericidal activity [26]. For instance, AgNPs have shown varying efficacy in inhibiting the growth of *S. epidermidis* [25]. Their antimicrobial action involves multiple pathways, including cell wall disruption, reactive oxygen species (ROS) generation, DNA interference, and the release of Ag⁺ ions [27, 28].

Similarly, zinc oxide nanoparticles (ZnO-NPs) exhibit strong antimicrobial effects through complex mechanisms. These include ROS production, the release of Zn²⁺ ions, electrostatic interactions, and the internalization of NPs into bacterial cells, which disrupts critical processes such as glycolysis, acid tolerance, and proton translocation [29, 30].

Recent septicemia outbreaks in fish have highlighted the significant economic consequences of *S. epidermidis* infections. Although *S. epidermidis* has been implicated in mass fish kills across diverse aquatic species, there remains a critical lack of in-depth studies on its molecular characterization, pathogenic mechanisms, and antimicrobial resistance (AMR) profiles in farmed fish, particularly tilapia (*Oreochromis* spp.). Furthermore, effective control strategies against *S. epidermidis* infections in aquaculture such as the application of silver nanoparticles (AgNPs) and zinc oxide nanoparticles (ZnO-NPs) remain underexplored. Consequently, The objectives of this study are threefold: first, to investigate the potential causes of mass fish kills associated with *S. epidermidis*; second, to conduct a molecular characterization (16 S rRNA) and assess the pathogenicity and antimicrobial resistance (AMR) of this bacterium; and third, to evaluate the in-vitro antimicrobial efficacy of silver nanoparticles and zinc oxide nanoparticles against *S. epidermidis*.

## Methods

### Sampling

**Fig. 1:**
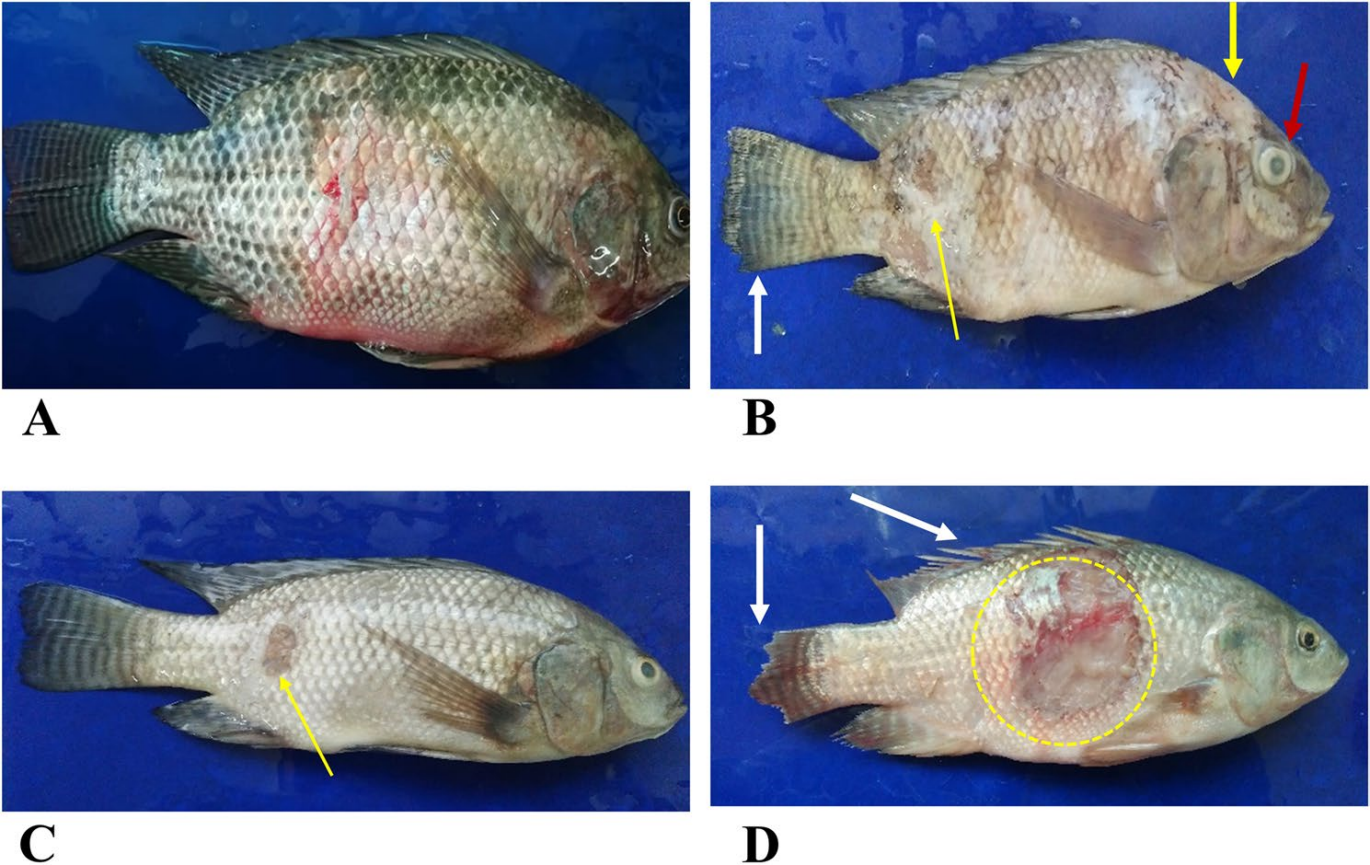
Adult tilapia naturally infected, exhibiting pronounced skin reddening and erosion **(A)**. Eye opacity (red arrow), along with patches of discoloration and extensive erosions (yellow arrow) and tail fin rot (white arrow) **(B)**. There is marked skin necrosis and ulceration (yellow arrow) **(C)**. A larger, deep ulceration extending into the musculature is shown within the yellow circle, along with tail fin rot (white arrow) and dorsal fin rot that exposes the dorsal fin rays (white arrow) **(D)**

Throughout the summer fish kill episode, a total of twenty-two cultured *Oreochromis* spp. (with an average weight of 180 ± 20 g) exhibiting clinical signs of the disease were collected from a semi-intensive earthen pond-based fish farms in Ismailia governorate, Egypt. The fish farm experienced substantial fish mortality, characterized by skin ulceration, tail fin rot and eye opacity (Fig. 1). Additionally, Some of the fish displayed scale loss, skin erosion, hemorrhages and dorsal fin rot exposing dorsal fin rays (Fig. 1). The cumulative mortality rates observed in diseased *Oreochromis* spp. were moderate, ranged between 20% and 25%. Additionally, the water conditions in the cultured pond included a temperature of 28 ± 3 °C, a pH level of 8.4 ± 0.2, dissolved oxygen concentration of 3.5 ± 0.5 mg/L, and ammonium-nitrogen levels measuring 3.0 ± 1.0 mg/L. The moribund fish were immediately placed in separate plastic bags containing water and kept under conditions of artificial aeration for live transport.

The fish samples were then quickly delivered to the Department of Aquatic Animal Medicine and Management, Faculty of Veterinary Medicine, Cairo University. Upon arrival, humane euthanasia was performed on all fish using an overdose of clove oil (≥ 98% eugenol, Sigma-Aldrich) at 150 mg/L (0.15 mL/L) [31]. To confirm death, the fish remained in the solution for 20 min following the cessation of opercular movement. Once euthanasia was confirmed, a series of examinations—including clinical, postmortem, and microbiological assessments—were conducted on the specimens.

Furthermore, all experimental protocols and methods adhered to the ARRIVE 2.0 standards (Animal Research: Reporting of In Vivo Experiments) guidelines. All procedures complied with the guidelines and regulations set by Veterinary Medicine, Cairo University Institutional Animal Care and Use Committee (Vet. CU. IACUC; Vet CU110520251097).

### Detection of viruses

#### Synthetic positive viral control

A synthetic positive control was designed in silico to simultaneously detect two viral pathogens in tilapia. The construct included a 605-nucleotide fragment of Nervous Necrosis Virus (NNV) RNA_2_ and a 484-nucleotide fragment of Tilapia Lake Virus (TiLV) segment 9, amplified using published primers [32, 33]. The entire synthetic fragment was subsequently cloned into a pBlueScript II SK(+) plasmid (Biomatik Corporation, Canada).

### Reverse Transcription-PCR (RT-PCR)

Internal organs including spleen, anterior kidney, and other organs (brain and eye) from diseased tilapia were processed for viruses detection. Total tissue RNA was extracted from brain, eye, anterior kidney and spleen of tilapia using RNeasy Mini Kit (Qiagen, Germany) according to the manufacturer’s instructions. Reverse transcription-PCR (RT-PCR) usingVerso 1-Step RT-PCR Kit ReddyMix (ThermoFisher, USA) was used to detect necrosis nervous virus (NNV) and tilapia lake virus (TiLV) in tissue RNA extracts according to Taha et al. [34]. Reverse transcription-PCR (RT-PCR) was performed in a 50 µl reaction volume. The thermal cycling protocol consisted of cDNA synthesis at 50 °C for 15 min, followed by reverse transcriptase inactivation at 95 °C for 2 min, 40 cycles of amplification (95 °C for 20 s, 46.5–57.5 °C for annealing [NNV or TiLV, respectively], and 72 °C for 1 min), and a final extension at 72 °C for 5 min. Each run included a non-template negative control (nuclease-free water) and a synthetic positive control. The resulting amplicons were visualized via electrophoresis on a 1.5% agarose gel stained with ethidium bromide. To confirm our results, we performed repeated RNA extraction and subsequent RT-PCR analysis on independent biological replicates.

### Isolation and identification of bacteria

Samples were taken from the skin lesion, kidney, spleen, brain, gills, eye and liver of moribund fish and were then inoculated aseptically on different culture media. The media used for bacterial isolation included Brain heart infusion agar (BHIA, Sigma-Aldrich), Nutrient agar (NA, Sigma-Aldrich), Blood agar (BA, HiMedia) supplemented with 5% sheep blood, MacConkey agar (Oxoid), Thiosulfate citrate bile salts sucrose agar (TCBS, Sigma-Aldrich), Salmonella Shigella agar (SS, Oxoid), Baird parker agar (BP, HiMedia) supplemented with egg yolk or egg yolk tellurite and Mannitol salt agar (MSA, HiMedia). The inoculated plates were incubated at 28 °C and 37 °C for 24–72 h. Bacterial colonies were obtained through streaking and re-streaking onto fresh media as previously mentioned, and only one type of colony was identified across all samples based on colony morphology and gram staining. Presumptive identification was carried out using different phenotypic [8] and biochemical tests (APIID Test Strips^®^, APIWEB™, Biomérieux, USA) in accordance with the manufacturer’s instructions. The presumptively identified pure cultures were subsequently stored at − 20 °C in Brain heart infusion broth (BHIB, Sigma-Aldrich) supplemented 16% glycerol (Sigma-Aldrich) for further characterization.

### Molecular characterization

The genomic DNA of bacterial isolates was extracted using the RNeasy Mini Kit (Qiagen, GmbH, Hilden, Germany) following to the manufacturer’s instructions. For the PCR amplification of the 16 S rRNA gene, universal primers were utilized: the forward primer (F: 5′-AGAGTTTGATCCTGGCTCAG-3′) and the reverse primer (R: 5′-GGTTACCTTGTTACGACTT-3′) [35]. The PCR reaction was conducted in a final volume of 50 µL, containing 25 µL of 2x DreamTaq^®^ Green Master Mix (Thermo Fisher Scientific, USA), 4 µL of DNA (50 ng/ µL) template, and 2 µL (10 nmol L^− 1^) of each primer. Amplification were carried out using a MyCycler™ thermal cycler (Bio-Rad, USA) under the following cycling conditions: initial denaturation at 95 °C for 10 min, followed by 35 cycles of denaturation at 95 °C for 30 s, annealing at 55 °C for 45 s, extension at 72 °C for 60 s, concluding with a final extension at 72 °C for 5 min. The amplified products were analyzed by electrophoresis on a 1.5% (W/V) agarose gel stained with ethidium bromide. Subsequently, the amplicons were sequenced in both directions using a 3500 genetic analyzer (Applied Biosystems™, USA) at Colors Medical Laboratories in Cairo, Egypt. The resulting sequences were compared against the NCBI database using BLAST^®^ [36] and were deposited in GenBank under the accession number MN153038. The neighbor joining algorithm in MEGA 12 was utilized to construct phylogenetic analysis using the Maximum Composite Likelihood method (Detailed methodology in supplementary file).

### Antimicrobial susceptibility

The sensitivity of *Staphylococcus epidermidis* (MN153038) isolate to antimicrobial agents was assessed by using Kirby-Bauer disk diffusion method on Muller-Hinton agar (Difco) [37]. Specifically, the antimicrobials tested included ampicillin (AM 10 µg), oxacillin (OX 1 µg) and amoxicillin (AX 25 µg), gentamicin (CN 10 µg), vancomycin (VA 30 µg), erythromycin (E 15 µg), cefoxitin (FOX 30 µg), fusidic acid (FD 10 µg), clindamycin (DA 10 µg), polymyxin B (PB 300 units), tetracycline (TE 30 µg), doxycycline (DO 30 µg), tigecycline (TGC 15 µg), oxolinic acid (OA 2 µg), flumequine (UB 30 µg), ciprofloxacin (CIP 30 µg), enrofloxacin (ENR 5 µg), levofloxacin (LEV 5 µg), nitrofurantoin (F 300 µg), novobiocin (NV 30 µg), florfenicol (FFC 30 µg), and trimethoprim/sulfamethoxazole (SXT 25 µg). Plates were incubated at 28 °C for 24 h. After the incubation period, the antimicrobial susceptibility to a total of 22 agents was determined in accordance with the guidelines set by the Clinical and Laboratory Standards Institute [38]. Antimicrobial susceptibility testing was performed in three independent replicates.

### Vancomycin susceptibility

Vancomycin hydrochloride (100 mg/mL in DMSO, Sigma-Aldrich) was employed to determine the minimum inhibitory concentration (MIC) using the standardized broth microdilution method, as recommended by the Clinical and Laboratory Standards Institute recommendation [38]. Briefly, a bacterial suspension was prepared to a density of 0.5 McFarland (10^8^ CFU/mL) from an overnight culture grown on brain heart infusion agar (BHIA, Sigma-Aldrich). Subsequently, this suspension was diluted 1000-fold in Mueller Hinton broth (Oxoid). A volume of 100 µL of the diluted bacterial suspensions (10^5^ CFU/mL) was then added into each well of microplate that contained 100 µL of Mueller Hinton broth with varying concentrations of vancomycin (0.125 µg/mL, 0.25 µg/mL, 0.5 µg/mL, 1 µg/mL, 2 µg/mL, 4 µg/mL, 8 µg/mL, 16 µg/mL, 32 µg/mL and 64 µg/mL). Following an incubation period of 24 h at 28 °C, the MIC for vancomycin was determined as the lowest concentration of the antibiotic that completely inhibited visible bacterial growth and interpreted according to MIC breakpoints established for *Staphylococcus* spp. other than *Staphylococcus aureus* [38]. Standard vancomycin-susceptible *Staphylococcus aureus* (hVSSA) strain ATCC 29,213 served as the negative control. Vancomycin Susceptibility was performed in three independent replicates.

### Experimental animals

A total of one hundred apparently healthy *Oreochromis* spp. fish, including juveniles and adults, were obtained from a semi-intensive earthen pond-based fish farms in Ismailia governorate, Egypt. In addition, one hundred and thirty healthy fingerlings were acquired from a hatchery located in Kafr El Sheikh governate, Egypt. The average body weight of thirty adult fish was 150 ± 20 g, while seventy juvenile fish averaged 30 ± 5 g. In contrast, the fingerlings had an average body weight of 2.30 ± 0.75 g.

Upon collection, The healthy fish were placed in separate 120-liter plastic containers (120 L) filled with water and maintained under conditions of artificial aeration for transport. The fingerlings were transported into plastic bags containing one-third fish and two-thirds oxygen. Subsequently, The fish were delivered to the Department of Aquatic Animal Medicine and Management, Faculty of Veterinary Medicine, Cairo University.

Upon arrival, the fish were kept in 500-liter tanks with aeration for two-week acclimation period. During this time, random samples were selected for thorough clinical, postmortem, and bacteriological analyses to ensure they were free from *Staphylococcus epidermidis*.

### Experimental challenge

Fish were randomized into groups in accordance with the ARRIVE 2 guidelines [39]. The strain of *Staphylococcus epidermidis* (MN153038) isolated during the study was utilized for the challenge tests. The bacterial suspension for intraperitoneal injection (IP) and one-hour immersion (Imm.) was prepared to concentrations of 3.0 × 10^8^ CFU/mL and 3.0 × 10^9^ CFU/mL, respectively, using plate counting methods and McFarland standards [8]. Detailed experimental design is provided in Table 1.

**Table 1:** Design of experimental challeng trials

Fish size	Group	# of fish	Dose and Application	Notes
Adult fish	Gp. 1 A	10	0.5 mL (i.p.), *S. epidermedis*	-
	Gp. 2 A	10	0.5 ml (IP), Sterile PBS	-
Juvenile fish	Gp. 1 J	10	0.1 mL (IP), *S. epidermedis*	-
	Gp. 2 J	10	0.1 ml (IP), Sterile PBS	-
	Gp. 3 J	10	(Imm.), water containing *S. epidermedis*	Scale removal
	Gp. 4 J	10	(Imm.), water	Scale removal
	Gp. 5 J	10	(Imm.), water containing *S. epidermedis*	-
	Gp. 6 J	10	(Imm.), water	-
Fingerlings	Gp. 1 F	20	(Imm.), water containing *S. epidermedis*	Scale removal
	Gp. 2 F	20	(Imm.), water	Scale removal
	Gp. 3 F	20	(Imm.), water containing *S. epidermedis*	-
	Gp. 4 F	20	(Imm.), water	-
	Gp. 5 F	20	Absolute control	-

*A* Adult fish, *J* Juvenile fish, *F* Fingerlings, *IP* Intraperitoneal injection, *PBS* Phosphate Buffer Saline, *Imm.* prolonged immersion for 1 h, scale removal: Scraping approximately 1.0 cm of the lateral body surface for juvenile fish and about 0.2 cm for fingerlings, absolute control: Fingerlings were kept unexposed to any experimental interference

Throughout the experiment, the fish were fed a commercial tilapia diet, with feed size and ratio optimized based on the size of the fish. The experimental challenges were conducted in 50-liter glass aquaria, which were aerated using air stones. The water quality parameters included a temperature of 28 ± 2 °C, a pH level of 7.5 ± 0.2, a dissolved oxygen concentration of 5.5 ± 0.5 mg/L, and ammonium-nitrogen levels of 0.6 ± 0.1 mg/L.

Fish were monitored daily for 21 days, with clinical signs and mortality recorded. The freshly dead fish were subjected to bacterial re-isolation to confirm Koch’s postulates. At the terminal point of the experiment, humane euthanasia of surviving fish was performed. This procedure involved immersion in a clove oil solution at 150 mg/L (0.15 mL/L) for 20 min [31]. after opercular movement had ceased, thereby ensuring death. For pathological analysis, tissue sampling was then conducted. Specifically, the target pathogen was aseptically retrieved from the kidney and cultured on Brain Heart Infusion Agar (BHIA) prior to its identification using standard phenotypic and biochemical methods.

### Characterization of AgNPs and ZnO-NPs

Silver nanoparticles (AgNPs) were obtained in sizes 10 nm and 100 nm, 0.02 mg/mL in an aqueous buffer stabilized with sodium citrate (Sigma-Aldrich). In addition, zinc oxide nanoparticles (ZnO-NPs) were utilized in powder form with an average particle size of 100 nm (TEM) (Sigma-Aldrich). All reagents were of analytical grade and used without further purification. Deionized water (18.2 MΩ cm^− 1^ at 25 °C) was obtained from a Milli-Q water purification system (Millipore, Darmstadt, Germany) and used for the preparation of all aqueous solutions.

The physicochemical properties of the synthesized nanoparticles were characterized using a suite of techniques. Firstly, the particle size distribution and zeta potential were analyzed using Dynamic Light Scattering (DLS) with a ZetaSizer NanoZS (Malvern Panalytical, UK).

To complement this data and examine the primary particle size and morphology, transmission electron microscopy (TEM) was performed using an EM900 instrument (Zeiss, Germany) operating at 80 kV, with a tungsten hairpin cathode and a wide-angle dual speed 2k CCD camera. For TEM sample preparation, a twenty-five-microliter aliquot of the nanoparticle solution was deposited onto graphite-coated copper grids and allowed to dry overnight before imaging. Subsequently, the average particle size was determined by analyzing at least 50 randomly selected nanoparticles from the TEM images using ImageJ^®^ software (version 1.54p).

Furthermore, the optical properties were assessed by measuring the absorption spectra with a UV-Vis spectrophotometer (NanoDrop 2000^®^, Thermo Fischer Scientific, USA) against a deionized water blank. Prior to measurement, the ZnO NP suspensions were sonicated for 10 min to ensure dispersion. The spectra were recorded over a wavelength range of 200–700 nm. Finally, to ensure reproducibility, all measurements were conducted at room temperature and repeated across three separate days.

**Fig. 2:**
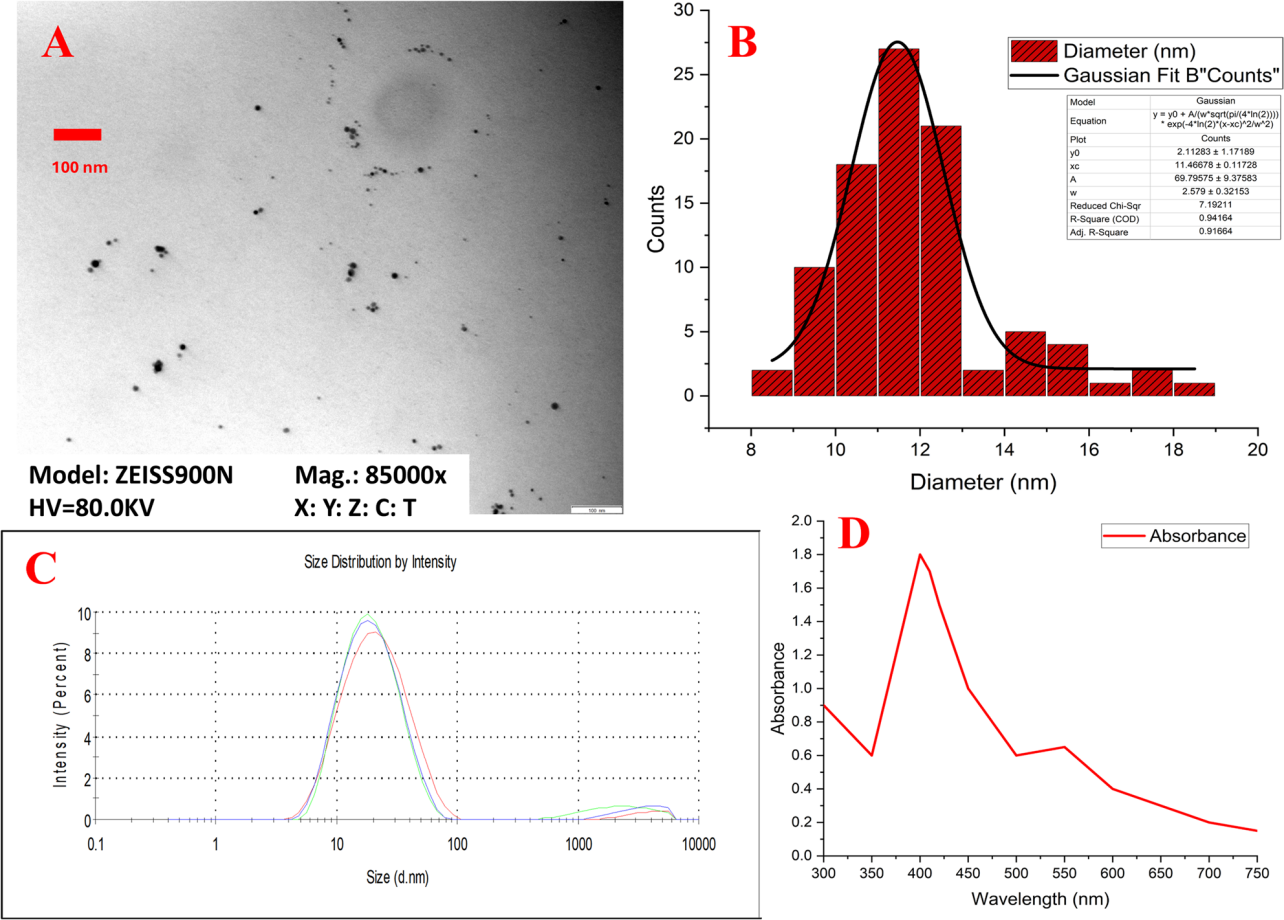
Characterization of silver nanoparticles (AgNPs, 10 nm) using transmission electron microscopy (TEM), dynamic light scattering (DLS) and UV-Vis spectroscopy. AgNPs were spherical in shape and had an average particle size of 11.47 nm **(A**,** B)**. The banner clearly displays TEM model (ZESIS900N), an accelerating voltage of 80.0 kV, and the magnification (85,000x). Scale bar represents 100 nm **(B)**. The particle size distribution of AgNPs indicated a monodisperse size distribution, peaking at 17.14 nm **(C)**. UV-Vis spectra of AgNPs exhibited a characteristic absorption maximum at 400 nm, corresponding to a surface plasmon resonance (SPR) value of 1.8 **(D)**

The silver nanoparticles (AgNPs, 10 nm) were predominantly spherical, with an average particle size of 11.47 ± 1.289 nm (Fig. 2A and B and supplementary Fig. 1). Additionally, they exhibited a unimodal size distribution, peaking at 17.14 nm (Fig. 2C), and had a zeta potential of − 25.7 mV. In UV-Vis spectroscopy, these AgNPs displayed a distinct absorption peak at 400 nm, corresponding to a surface plasmon resonance (SPR) peak of 1.8 (Fig. 2D). On the other hand, the detailed characterization of larger AgNPs (100 nm) and zinc oxide nanoparticles (ZnO-NPs, 100 nm) have been previously reported by Abou-Okada et al. [21], confirming a successful synthesis and detailed characterization. Specifically, 100 nm AgNPs were spherical, with an average size of 102.50 ± 2.35 nm, a unimodal distribution (peak at 100.5 nm), and a zeta potential of − 42.0 mV. Their UV-Vis spectrum showed an absorption peak at 400 nm and an SPR peak of 1.53. In contrast, the ZnO-NPs had a cylindrical (rod-like) morphology, with an average size of 110.27 ± 27.46 nm, a monomodal distribution (peak at 212.3 nm), and a positive zeta potential of 31.7 mV. Their optical properties differed significantly, with an absorption peak at 370 nm and an SPR peak of 4.0 [21].

### Antimicrobial activity of AgNPs and ZnO-NPs

To assess the antimicrobial efficacy of AgNPs and ZnO-NPs, the broth microdilution method was employed, following the guidelines set forth by the Clinical and Laboratory Standards Institute [38]. The minimal inhibitory concentration (MIC) for AgNPs (10 nm, and 100 nm) and ZnO-NPs (100 nm) was evaluated against *S. epidermidis* (MN153038) in BHI broth medium. Tests were conducted using disposable 96-well microtitration plates, where 100 µL of diluted bacterial suspensions (10^5^ CFU/mL) were added to each well containing 100 µL of varying concentrations of AgNPs (0.156 µg/mL, 0.3125 µg/mL, 0.625 µg/mL, 1.25 µg/mL, 2.5 µg/mL, 5 µg/mL, 10 µg/mL and 20 µg/mL) or ZnO-NPs (7.8 µg/mL, 15.6 µg/mL, 31.25 µg/mL, 62.5 µg/mL, 125 µg/mL, 250 µg/mL, 500 µg/mL and 1000 µg/mL). The negative control consisted solely of *S. epidermidis* without any nanoparticles. Following this, the microtitration plates were incubated at 28 °C for up to 48 h, after which the MIC for both AgNPs and ZnO-NPs was determined. The MIC was defined as the lowest concentration of nanomaterials that visibly inhibited 99% of *S. epidermidis* growth. The minimum inhibitory concentration (MIC) test of nanoparticles was performed in three independent replicates.

Subsequent to determining the MIC of AgNPs and ZnO-NPs, 50 µL aliquots from all tubes exhibiting no visible bacterial growth were streaked onto BHIA plates. These plates were then incubated at 28 °C for up to 48 h. The minimal bactericidal concentration (MBC) was identified as the lowest concentration of nanomaterials at which 99.9% of *S. epidermidis* population was completely inhibited.

### Statistical analysis

Statistical analysis was performed using GraphPad Prism version 8.2.0 (GraphPad Software, Boston, Massachusetts, USA). The normality of residuals and homogeneity of variances were assumed using the Shapiro-Wilk test and Levene’s test, respectively. For survival analysis, Kaplan-Meier survival plots were generated, and survival rates were compared using the log-rank test. Data are presented as mean ± standard error. Statistical significance between the zones of inhibition (mm) for different antimicrobials was determined using a one-way ANOVA with Tukey’s post-hoc test for pairwise comparisons. A p-value of < 0.05 was considered statistically significant.

## Results

### Isolation and identification of the causative agent

**Fig. 3:**
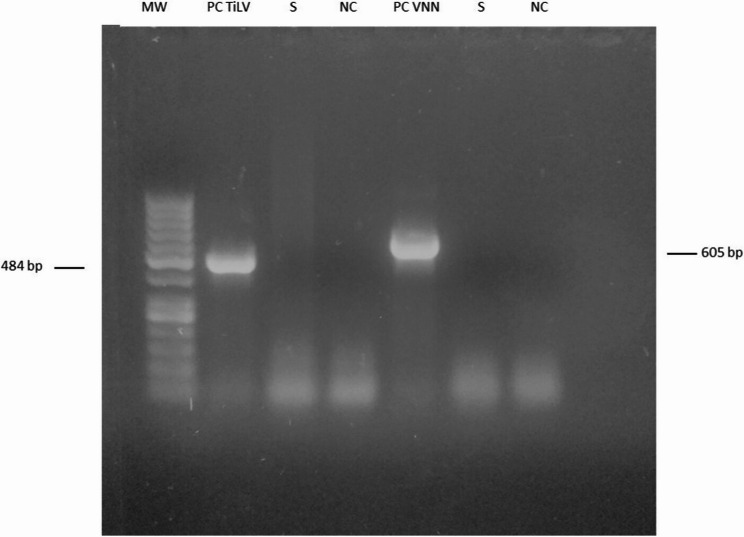
No detection of Tilapia lake virus (TiLV) RNA and Nervous necrosis virus (NNV) RNA in tissues of diseased tilapia. MW: GeneRuler® 50 bp DNALadder (Thermo Fisher Scientific™), S: sample, NC: non-template negative control, PC: Positive control, TiLV: Tilapia lake virus, and VNN: virus nervous necrosis. Uncropped full-length gels are presented in Supplementary Figs. 1 and 2

Tilapia lake virus (TiLV) RNA and Nervous necrosis virus (NNV) RNA were not detected in total RNA extracts of brain, eye, spleen, and anterior kidney of diseased tilapia (Fig. 3, Supplementary Fig. 2, 3). To confirm our results, we performed repeated RNA extraction and subsequent RT-PCR analysis on independent biological replicates. In all replicate samples, the results remained negative for both viruses. The isolated bacterial colonies appeared as small (1–2 mm) white raised spherical colonies on BHIA and NA plates at both 28 °C and 37 °C. In contrast, no growth was observed on TCBS, SS agar, R-S agar, or MacConkey agar at these temperatures. Additionally, on blood agar enriched with 5% sheep blood, non-hemolytic white raised colonies measuring 1–2 mm were noted. On mannitol salt agar, red colonies of similar size (1–2 mm) were found, while a 1-mm black colony with no clear zone was present on Baird Parker agar supplemented with egg yolk tellurite. The isolated bacteria were identified as Gram-positive cocci, appearing either as irregular grape-like clusters or as single cells under light microscope. The phenotypic and biochemical characteristics of these isolates are listed in Table 2. Ultimately, all isolates were classified as *S. epidermidis* based on their phenotypic and biochemical profiles.

**Table 2:** Phenotypic and biochemical characteristics of *Staphylococcus epidermidis* isolated from tilapia spp

Characteristics	Result	Characteristics	Result
Growth on:		Growth at:	
BHIA	+	28 °C	+
BP	+	37 °C	+
MSA	+	Grain stain	+
TCBS	-	Morphology	Spherical cocci
SS agar	-	Motility	-
MacConkey agar	-	Glucose	+
Blood agar	Non-hemolytic	Mannose	+
NA + 0% NaCl	+	Inositol	-
NA + 2% NaCl	+	Sorbitol	-
NA + 4% NaCl	+	Rhamnose	-
NA + 6% NaCl	+	Sucrose	+
NA + 8% NaCl	+	Melibiose	-
NA + 10% NaCl	+	Amygdalin	-
NA + 12% NaCl	+	Arabinose	-
Production of:		Mannitol	-
Oxidase	-	Maltose	+
Catalase	+	Lactose	-
Slide coagulase	-	Esculin	-
Tube coagulase	-	Lysine decarboxylase	-
Urease	+	ß-galactosidase	-
H_2_S	-	Arginine DiHydrolase	-
Indole	-	Ornithine DeCarboxylase	-
Voges Proskauer	+	Citrate utilization	-
Methyl red	+	Tryptophane DeAminase	-
Nitrate reduction	+	Gelatinase	-

+Positive reaction

-Negative reaction

### Molecular characterization

**Fig. 4:**
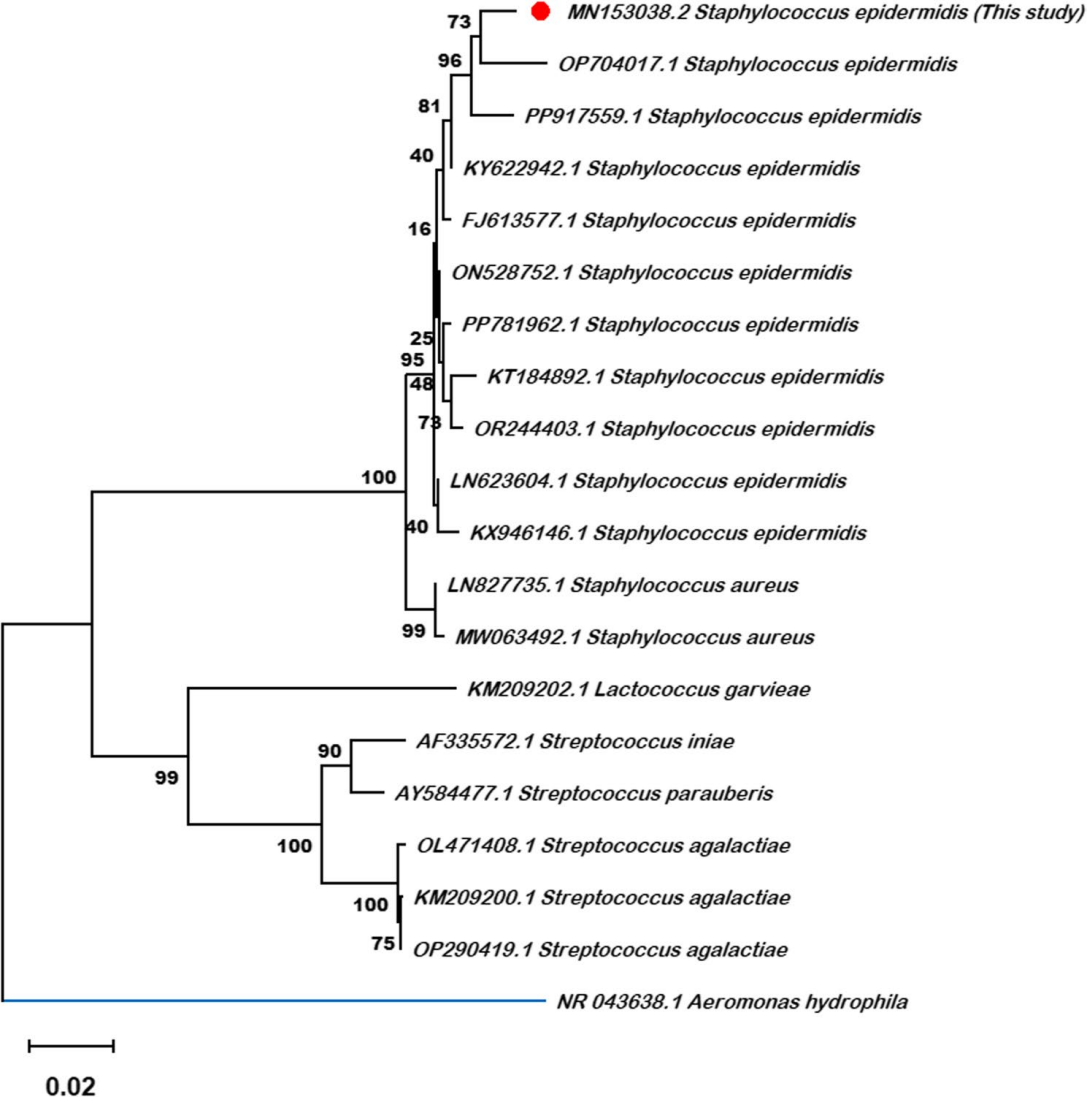
The analytical procedure encompassed 20 nucleotide sequences. The pairwise deletion option was applied to all ambiguous positions for each sequence pair resulting in a final data set comprising 1,636 positions. Evolutionary analyses were conducted in MEGA12 utilizing up to 7 parallel computing threads. The bootstrap values (%) are displayed next to the clades, and the accession numbers are indicated before the strain names. The tree was rooted to Aeromonas hydrophila as the outgroup. The bootstrap values (given as a percentage of 1000 replicates) are displayed at each branch nod (scale bar = 0.02 substitutions per nucleotide)

The pure cultures of *Staphylococcus epidermidis* were confirmed by sequencing 16 S rRNA region. The 1147 bp 16 S rRNA gene of *S. epidermidis* (accession number MN153038) demonstrated 100% coverage and over 96% identity with *S. epidermidis* isolates (LN623604, KX946146, PP917559, ON528752, FJ613577, KT184892, & PP781962). Notably, the accession number MN153038 represents the first sequence of *S. epidermidis* isolated from tilapia species during the summer fish kills, with the sequence being submitted to GenBank in March 2020. Additionally, a phylogenetic tree was constructed utilizing 16 S rRNA gene sequences (Fig. 4). The analysis revealed that the bacterial isolate from this study (MN153038) was placed in a single clade with confirmed *S. epidermidis* sequences. Consequently, this phylogenetic affiliation provides robust, standard confirmation of the isolate’s species identity.

### Antimicrobial susceptibility

The antimicrobial susceptibility profile of the Staphylococcus epidermidis isolate was determined by disk diffusion assay **(**Table 3**)**. The isolate was resistant to 12 antimicrobial agents, including oxacillin, ampicillin, amoxicillin, gentamicin, erythromycin, cefoxitin, fusidic acid, clindamycin, tetracycline, polymyxin B, oxolinic acid, and flumequine. In contrast, susceptibility was observed to 10 agents, including florfenicol, tigecycline, doxycycline, ciprofloxacin, enrofloxacin, levofloxacin, nitrofurantoin, novobiocin, sulfamethoxazole/trimethoprim, and vancomycin.

**Table 3:** Sensitivity of *Staphylococcus epidermidis* isolate to 22 antimicrobials

Antimicrobials	Concentration	Inhibition zone (mm) Mean ± SE	Sensitivity
Ampicillin	10 mcg	0.00 ± 0.000^**j**^	R
Oxacillin	1 mcg	15.00 ± 0.265^**g**^	R
Amoxicillin	25 mcg	8.00 ± 0.132^**i**^	R
Gentamicin	10 mcg	11.00 ± 0.153^**h**^	R
Vancomycin	30 mcg	18.00 ± 0.200^**e**^	S
Erythromycin	15 mcg	0.00 ± 0.000^**j**^	R
Cefoxitin	30 mcg	12.00 ± 0.058^**h**^	R
Fusidic acid	10 mcg	12.00 ± 0.252^**h**^	R
Clindamycin	10 mcg	14.00 ± 0.000^**g**^	R
Polymyxin B	300 unit	16.00 ± 0.098^**f**^	R
Tetracycline	30 mcg	14.00 ± 0.100^**g**^	R
Doxycycline	30 mcg	21.00 ± 0.058^**d**^	S
Tigecycline	15 mcg	23.00 ± 0.577^**c**^	S
Oxolinic acid	2 mcg	15.00 ± 0.115^**g**^	R
Flumequine	30 mcg	18.00 ± 0.289^**e**^	R
Ciprofloxacin	30 mcg	25.00 ± 0.153^**b**^	S
Enrofloxacin	5 mcg	30.00 ± 0.100^**a**^	S
Levofloxacin	5 mcg	32.00 ± 0.173^**a**^	S
Nitrofurantoin	300 mcg	20.00 ± 0.289^**d**^	S
Novobiocin	30 mcg	34.00 ± 0.000^**a**^	S
Florfenicol	30 mcg	25.00 ± 0.115^**b**^	S
Sulfamethoxazole /trimethoprim	25 mcg	23.00 ± 0.200^**c**^	S
*F (df*_*1*_, *df*_*2*_*)*		**2021 (21**,** 44)**	
*P value*		**0.00001**	

*mcg* Microgram, *R* Resistant, *S* Sensitive, data are presented as mean ± SE, SE: standard error of mean (*n* = 3). Statistically significant differences were observed at *p* < 0.05 (ANOVA, Tukey’s post hoc)

However, the disk diffusion method cannot reliably differentiate between vancomycin-susceptible, -intermediate, and -resistant phenotypes due to overlapping inhibition zone diameters. Therefore, minimum inhibitory concentration (MIC) testing was employed to accurately define vancomycin susceptibility.

### Vancomycin susceptibility

*Staphylococcus epidermidis* isolate exhibited susceptibility to vancomycin, as determined by a mean minimum inhibitory concentration (MIC) of 4 µg/mL. At this concentration, vancomycin achieved complete bacterial growth inhibition.

### Experimental challenge

**Fig. 5:**
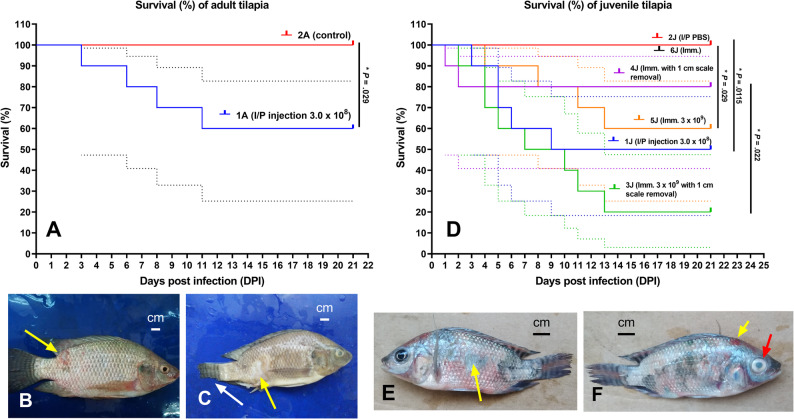
Survival rate (%) of adult tilapia 21 days post-infection with *Staphylococcus epidermidis* **(A)**. Adult tilapia displaying skin reddening and erosion (yellow arrow) **(B)**. Adult tilapia displaying patches of discoloration and erosion (yellow arrow), along with tail fin rot (white arrow) **(C)**. Survival rate (%) of juvenile tilapia 21 days post-infection with *Staphylococcus epidermidis* **(D)**. Juvenile tilapia displaying skin reddening, patches of discoloration and erosion (yellow arrow), along with tail fin rot **(E)**. Juvenile tilapia displaying skin hemorrhage (yellow arrow), along with eye opacity (red arrow) **(F)**

**Fig. 6:**
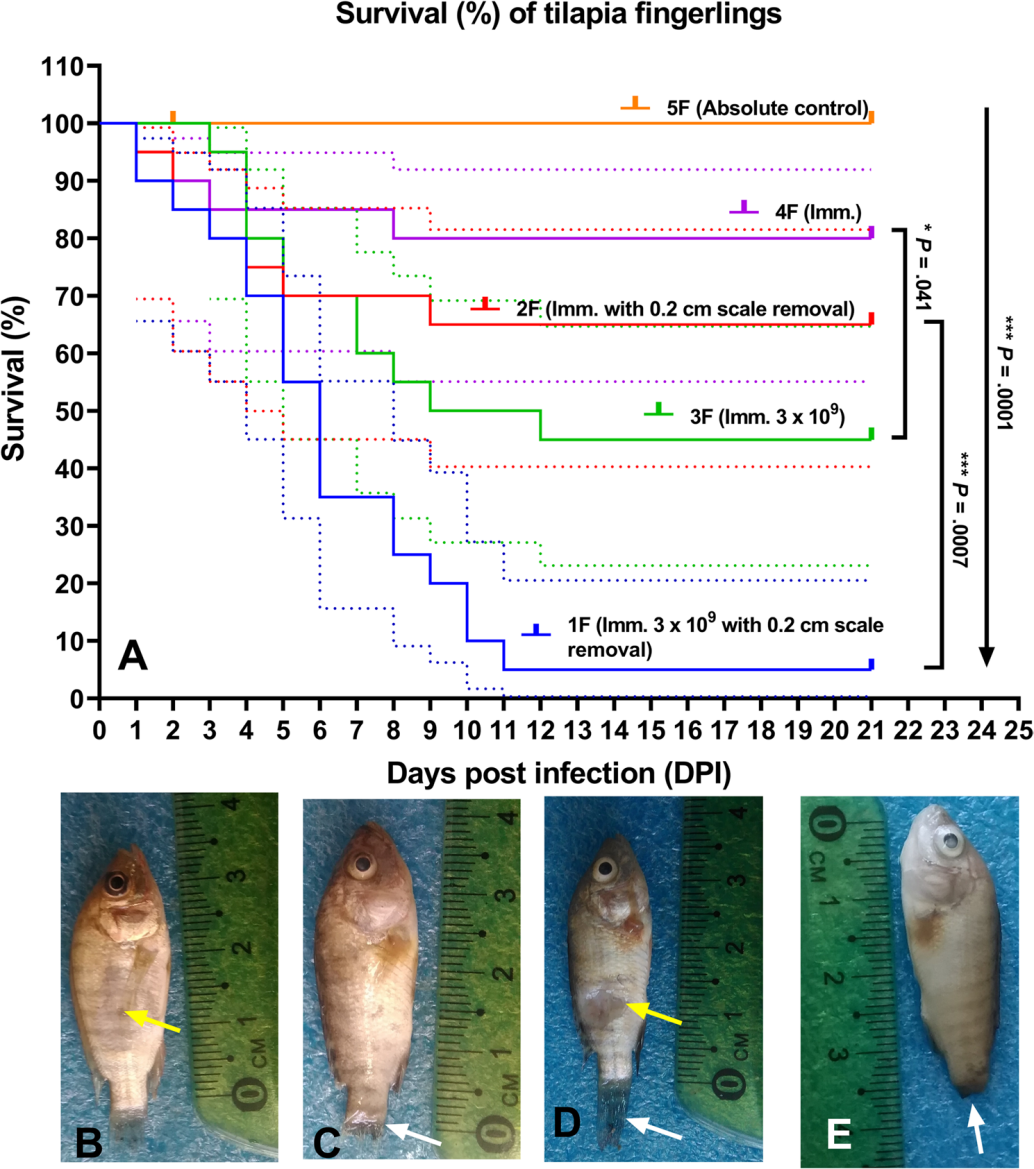
Survival rate (%) of tilapia fingerlings 21 days post-infection with *Staphylococcus epidermidis* **(A)**. Fingerling showing patches of discoloration and erosion (yellow arrow) **(B)**. Fingerling showing severe tail fin rot (white arrow) **(C)**. Fingerlings showing tail fin rot (white arrow) along with a larger, deep ulceration extending into the musculature (yellow arrow) **(D)**. Fingerling showing cloudy eyes and a complete loss of its tail fin (white arrow) **(E)**

The results of the pathogenicity tests are listed in Figs. 5 and 6. Various factors, including administration methods, dosage and tilapia size, influence the survival rate of the challenged fish. In adult fish (150 ± 20 g) injected intraperitoneally (IP) with 0.5 mL *S. epidermidis* at concentration of 3.0 × 10^8^ CFU/mL, mortality began on the third day post-infection, culminating in a cumulative mortality rate of 40%. Conversely, juvenile fish (30 ± 5 g) that received a 0.1 mL IP injection of the same pathogen at the same dose experienced mortality starting on the third day, with a cumulative mortality of 50%.

For juvenile fish subjected to a 1-hour immersion challenge at a dose of 3.0 × 10^9^ CFU/mL with 1-cm scale removal, the mortality commenced on the second day, 80% cumulative mortality. In contrast, the group without scale removal experienced mortality starting on the fourth day, resulting in a cumulative rate of 40%. Additionally, fingerlings (2.30 ± 0.75 g) that underwent a 1-hour immersion with a dose of 3.0 × 10^9^ CFU/mL and 0.2-cm scale removal exhibited mortality beginning on the first day, with a cumulative rate of 95%. In the group without scale removal, mortality started on the third day, culminating in a cumulative mortality of 55%.

In the control group that underwent scale removal and a 1-hour immersion in water, cumulative mortality rates reached 20% for juvenile fish (Gp. 4 J) and 35% for fingerlings (Gp. 2 F). Notably, no mortality was observed in the control group without scale removal or in the control group that received IP injection of sterile phosphate-buffered saline (PBS). The survival rate (%) of adult, juvenile and fingerling tilapia are illustrated in Figs. 5A and D, and 6A, respectively.

The clinical signs observed in experimental fish infected with *S. epidermidis* infection (MN153038) were similar to those seen in naturally infected fish. In adult fish, the signs included skin hemorrhage, patches of discoloration, ulceration, and tail fin rot (Fig. 5B and C). Meanwhile, juvenile fish exhibited skin hemorrhage, erosion, ulceration, eye opacity, and tail fin rot (Fig. 5E and F). In fingerlings, the clinical signs were more severe, featuring larger, deep ulceration that extended into the musculature, cloudy eyes, and complete loss of the tail fin (Fig. 6B, C and D, and E).

*Staphylococcus epidermidis* was successfully re-isolated from the kidney of experimentally infected fish. The re-isolated bacteria were confirmed to be identical to the original challenge strain (MN153038) through comparative analysis. This was based on congruent colony morphology, identical biochemical profiles, and a 100% sequence match of the 16 S rRNA gene, thereby fulfilling Koch’s postulates. In contrast, no bacteria were recovered from the control fish.

### Antimicrobial activity of AgNPs and ZnO-NPs

A comparative analysis of the antimicrobial efficacy of silver nanoparticles (AgNPs) and zinc oxide nanoparticles (ZnO-NPs) against *Staphylococcus epidermidis* demonstrated significant variations, which were primarily dependent on nanoparticle composition and size. Specifically, the antimicrobial activity, as determined by minimum inhibitory concentration (MIC) values, revealed that 10 nm AgNPs were substantially more potent than their 100 nm AgNP counterparts and 100 nm ZnO-NPs. For example, 10 nm AgNPs exhibited a strong inhibitory effect with a mean MIC of 1.25 µg/mL. In contrast, the mean MIC for 100 nm AgNPs was considerably higher at 10 µg/mL, suggesting lower antimicrobial efficacy. Furthermore, ZnO-NPs displayed the highest mean MIC of 125 µg/mL, indicating the weakest inhibitory action among the tested nanoparticles.

Moreover, this trend was consistently supported by the minimum bactericidal concentration (MBC) results. The 10 nm AgNPs demonstrated a mean MBC of 2.5 µg/mL, confirming their capacity to fully eradicate the bacterial cells. Conversely, a significantly higher mean MBC of 20 µg/mL was observed for the 100 nm AgNPs, while the mean MBC for ZnO-NPs was the highest, at 250 µg/mL.

## Discussion

Using RT-PCR, neither Tilapia lake virus (TiLV) nor Nervous necrosis virus (NNV) RNA was detected in any of the tested extracts. In this study, the cumulative mortality rates in diseased *Oreochromis* spp. ranged between 20% and 25%. In contrast, Taha et al. [34] identified NNV RNA in diseased larvae exhibiting abnormal swimming behavior and eye opacity, with cumulative mortality rates reaching up to 70%.

Nervous necrosis virus (NNV, Betanodavirus) has a broad host range, infecting more than 120 fish species globally [40]. While it primarily affects marine fish, inducing severe neuropathological conditions with high mortality and morbidity rates [41, 42], research has confirmed its presence in freshwater environments as well. In fact, several studies have reported NNV infections in various freshwater fish species [43, 44], demonstrating that the virus can adapt to different aquatic ecosystems. Notably, The virus targets the brain, spinal cord, central nervous system (CNS), and retina of larval and juvenile fish, frequently resulting in 100% mortality during outbreaks [40, 41, 43]. However, NNV detections in adult tilapia are uncommon [45], which is consistent with our findings, as no NNV RNA was detected in this study. Nevertheless, isolated cases have been reported [34, 44], suggesting that while rare, NNV can still affect adult tilapia under certain conditions.

Tilapia lake virus (TiLV, Tilapinevirus) represent a serious global threat to tilapia populations, as this highly contagious pathogen causes tilapia lake virus disease (TiLVD) with devasting effects. Outbreaks typically result in severe illness and mass mortality in both cultured and wild tilapia across multiple continents [46–48]. Research conducted by Fathi et al. [49] demonstrated the virus’s significant presence, with RT-PCR testing confirming TiLV infections in tilapia from three out of seven farms experiencing summer mortality syndrome. Furthermore, Mugimba et al. [50] identified TiLV through RT- PCR in both cultured and wild Nile tilapia from Lake Victoria, marking the first confirmed detection in these populations.

TiLV and NNV RNA were not detected in this study; therefore, the observed disease symptoms, including lethargy, skin ulceration, tail fin rot, eye opacity, and mortality, likely stemed from other pathogens [45, 51]. Notably, bacterial infections are a major contributor to disease outbreaks in tilapia, often resulting in severe economic losses worldwide. Several bacterial pathogens have been frequently associated with tilapia diseases, such as *Flavobacterium columnare* [52], *Aeromonas* spp [53, 54], *Streptococcus* spp [55] and *Staphylococcus* spp [9].

In this study, Gram-positive cocci were successfully isolated from diseased tilapia. The bacterial isolates displayed highly similar phenotypic and biochemical profiles, which were initially classified as *Staphylococcus epidermidis*. To ensure accurate identification, the results were further validated through 16 S rRNA gene sequencing (GenBank accession: MN153038). Importantly, the combined application of phenotypic, biochemical, and molecular techniques especially 16 S rRNA sequencing that substantially enhances the reliability of bacterial characterization [56]. Indeed, DNA sequence-based analysis is widely regarded as the gold standard for identifying coagulase-negative staphylococci (CoNS), owing to its superior taxonomic accuracy [57].

Although *S. epidermidis* is not commonly recognized as a fish pathogen, previous studies have documented its role as a pathogen in diverse aquatic species, including freshwater, brackish, and marine fish species in Egypt, Japan, Turkey, Taiwan, Greece, and Iran [7, 10–13]. Notably, it has also been implicated in infections affecting tilapia (*Oreochromis* spp.) [8, 9]. *S. epidermidis* was confirmed by satisfying Koch’s postulates. Experimentally infected fish developed clinical disease, and *S. epidermidis* was re-isolated from moribund and dead fish, thereby solidifying its causative role.

*Staphylococcus epidermidis* displayed a worrisome multidrug resistance (MDR) profile, demonstrating resistance to twelve antimicrobial agents while remaining susceptible to ten others, offering potential therapeutic alternatives. Notably, vancomycin exhibited strong efficacy, completely inhibiting bacterial growth at a concentration of 4 µg/mL. These findings underscore the growing challenge of antimicrobial resistance (AMR) in *S. epidermidis*. Although the susceptibility to certain drugs provides clinical options, their judicious use is critical to curb further resistance development [19, 20].

This resistance pattern aligns with previous reports documenting *S. epidermidis* resistance to penicillin, ampicillin, ampicillin-sulbactam, methicillin/oxacillin, kanamycin, cefoxitin, erythromycin, gentamycin, oxytetracycline, streptomycin, clindamycin, tobramycin, and oxacillin [12, 17]. Of particular concern is the ability of staphylococci, especially coagulase-negative staphylococci (CoNS), to form biofilms—a key factor in persistent infections [58, 59]. Biofilms not only enhance bacterial survival in aquatic environments but also promote the exchange of mobile genetic elements, accelerating the spread of resistance genes among aquatic bacteria [60]. Furthermore, biofilm formation shields pathogenic bacteria from host defenses and antibiotic treatments, complicating clinical management [58]. Genomic analyses reveal that *S. epidermidis* carries numerous virulence and antibiotic resistance genes, some of which are shared with *S. aureus*, suggesting horizontal gene transfer between these species [61]. Specifically, strains *S. epidermidis* MDH2 and MDH5 exhibited resistance to at least three antimicrobial classes, yet showed no phenotypic resistance to ciprofloxacin, tetracycline, florfenicol, or vancomycin [17].

*Staphylococcus epidermidis* is recognized as a pathogenic bacterium affecting fish in freshwater, brackish, and marine environments globally. Notably, outbreaks of this pathogen have led to mass mortalities in tilapia (*Oreochromis* spp.), as reported in previous studies [8]– [9, 62]. Furthermore, in the current study, *S. epidermidis* was found to induce clinical disease signs in tilapia across different life stages, including fingerlings, juveniles, and adults. Moreover, the mortality rates varied significantly, ranging from 40% to 95%, depending on factors such as the administration method, bacterial dosage, and the size of the infected fish.

Experimental infections of fish with *S. epidermidis* consistently produce clinical signs that mirror those observed in natural outbreaks and previous studies. For instance [8], reported that severely infected tilapia developed characteristic signs such as epidermal lesions, fin ulceration, and, in some cases, exophthalmia. Similarly, Kusuda and Sugiyama [6] described comparable pathological changes, including skin congestion, tail ulcerations, and exophthalmia, in infected yellowtail and red sea bream. However, species-specific variations in disease presentation have also been documented. Kubilay and Uluköy [12] found that gilthead sea bream infected with *S. epidermidis* primarily exhibited lethargy, hemorrhagic lesions on the fins and mouth, and dark skin discoloration. Likewise, Metin et al. [14] observed comparable signs in trout, including hemorrhagic eyes and jaws, along with pronounced darkening of the skin.

Importantly, the intensity and progression of these clinical manifestations are influenced by multiple factors, including fish size, bacterial load, administration route, and concurrent stress conditions. Nevertheless, regardless of these variables, *S. epidermidis* infections consistently result in high mortality rates, leading to severe economic consequences for aquaculture operations [6, 8, 11–14].

Tilapia (*Oreochromis* spp.) are generally considered more resilient against common pathogens than many other fish species [63]. However, recent studies indicate that intensively farmed tilapia populations are becoming increasingly vulnerable to various pathogenic organisms [64]. In the present, stressed fingerling tilapia exhibited significantly higher susceptibility to *S. epidermidis* infections compared to juveniles and adults. This vulnerability stems from their underdeveloped immune systems and reduced stress tolerance [65]. Furthermore, stress—whether from poor water quality, overcrowding, or handling—compromises immune function by elevating cortisol levels, which suppresses both innate and adaptive immune responses [13, 66]– [67].

The skin constitute the first line of immunological defense in fish, serving as critical interfaces that prevent pathogen invasion [68]– [69]. The fish skin employs multiple synergistic defense strategies against *S. epidermidis* invasion. The skin’s stratified structure forms an effective physical barrier that hinders bacterial adhesion and penetration, while its mucus secretion contains potent antimicrobial compounds such as piscidins and lysozymes that actively combat potential pathogens [70]. Complementing these mechanisms, the skin’s indigenous microbiota provides ecological protection by outcompeting pathogenic bacteria for resources and colonization sites [64, 71]. This multilayered protection is essential for maintaining piscine health in pathogen-rich aquatic ecosystems.

Aquaculture environments frequently expose fish to stressful conditions that can disrupt their microbial balance, promoting the propagation of opportunistic pathogens [72, 73]. *Staphylococcus epidermidis*, a common component of the aquatic microbiome, becomes particularly problematic when fish experience skin damage from scale loss, abrasions, or handling injuries. Under these compromised conditions, the bacteria can penetrate deeper tissues, causing severe infections such as skin ulceration, fin rot, and ultimately high mortality rates [8, 62]. Through controlled immersion challenges with *S. epidermidis*, our study provides experimental validation of skin barrier function in tilapia, revealing dramatically higher mortality rates in scale-compromised fish (80% in fingerlings, 95% in juveniles) compared to those with intact skin (40% and 55% mortality, respectively). These results clearly demonstrate that intact skin serves as a vital defensive barrier, emphasizing its critical role in tilapia immunity and overall survival in aquaculture operations [68, 70].

*Staphylococcus epidermidis* demonstrates significant pathogenicity in tilapia through multiple interconnected virulence mechanisms. Slime production initially recognized as a critical factor in 1972 [74], biofilm formation serves as the cornerstone of its virulence by enabling surface adhesion while simultaneously providing protection against both antimicrobial agents and host immune defenses [75]. Beyond structural advantages, the pathogen employs biochemical offensive strategies through the secretion of destructive enzymes including proteases, lipases, and elastase-active metalloproteases, complemented by cytotoxic compounds such as δ-toxin [76, 77]. These virulence factors operate synergistically, while δ-toxin directly compromises cellular integrity by disrupting membranes, extracellular enzymes systematically degrade host tissues, collectively resulting in extensive tissue damage and apoptosis [78]. The situation is particularly exacerbated in aquatic environments where biofilm resilience on mucosal surfaces facilitates persistent infections that resist conventional treatment approaches [75], demonstrating the organism’s remarkable adaptability and pathogenic complexity in aquatic hosts.

Antimicrobial resistance (AMR) in aquaculture is a critical global issue, particularly because aquatic foods such as fish and shellfish serve as a major source of animal protein in both developed and developing nations [18]. To address this challenge, several alternatives to antimicrobials have been proposed, including prebiotics, probiotics, immunostimulants, vaccines, essential oils (EOs), peptides, phage therapy, and nanoparticles. These alternatives not only help reduce the risk of AMR but also enhance the health and productivity of aquatic species [18, 21, 79]– [80]. Among these, metallic and metallic oxide nanoparticles (MONPs) have gained attention due to their unique properties, which distinguish them from conventional antibiotics and bulk materials. Specifically, MONPs have shown promise as effective antibacterial agents against multidrug-resistant (MDR) Gram-positive and Gram-negative bacteria [81]. Notably, silver nanoparticles (AgNPs) act as broad-spectrum biocides, targeting various drug-resistant pathogens [82], while both AgNPs and zinc oxide nanoparticles (Zn-ONPs) demonstrate strong antimicrobial effects against Gram-positive bacteria such as *S. epidermidis* [25, 55]. Importantly, metal nanoparticles (MNPs) exhibit a lower likelihood of inducing bacterial resistance compared to traditional antibiotics [81], making them a viable long-term solution for sustainable aquaculture practices.

Substantial evidence has established the anti-staphylococcal potency of silver nanoparticles (AgNPs), with sizes ranging from 10 nm to 100 nm, at concentrations between 1 and 10 µg/mL [25]. In line with this previous work, the present study also demonstrated the bactericidal activity of AgNPs (10–100 nm) and zinc oxide nanoparticles (ZnO-NPs, 100 nm) against the Gram-positive bacterium *S. epidermidis*. A critical finding from our investigation was that the antibacterial efficacy was not uniform but varied significantly based on several factors, including nanoparticle type, concentration, particle size, and the biofilm-forming capacity of the bacterial strain.

Specifically, our results corroborate the findings of Swolana et al. [25], who reported that AgNPs (10–100 nm) inhibited *S. epidermidis* growth at minimum inhibitory concentrations (MICs) of 1–5 µg/mL. However, a notable discrepancy emerges when compared to the study of Sheikholeslami et al. [83], who determined a considerably higher minimum bactericidal concentration (MBC) of 62.5 µg/mL for AgNPs (~ 40 nm) against the same species. This divergence in effective concentrations may be attributed to differences in experimental protocols or nanoparticle synthesis methods.

Furthermore, our analysis supports the observed mechanism that AgNPs display stronger antimicrobial activity against biofilm-forming strains, which is principally attributed to their accumulation within the biofilm matrix. Conversely, smaller AgNPs were found to be more effective against non-biofilm-forming strains [25], suggesting that particle size is a critical determinant of the interaction mechanism with different bacterial phenotypes.

Silver nanoparticles (AgNPs) exert their antimicrobial effects through the sustained release of silver ions (Ag⁺) in aqueous environments, with their enhanced bactericidal activity attributed to their larger surface area [84]. The mechanism of action involves direct interaction between AgNPs and bacterial enzymatic systems, triggering reactive oxygen species (ROS) generation. Elevated ROS levels induce oxidative stress, leading to protein and nucleic acid damage [27]. Additionally, AgNPs accumulate on bacterial cell walls, forming cavities and indentations that compromise membrane integrity and permeability. This disruption impairs essential cellular functions, including respiration, ultimately resulting in apoptosis [25, 85]. However, bacterial susceptibility to AgNPs varies depending on pathogen type. Gram-negative bacteria are generally more vulnerable than Gram-positive species due to structural differences in their cell walls; Gram-positive bacteria possess thick peptidoglycan layers that hinder nanoparticle penetration [27]. Notably, Gram-negative bacteria exhibit approximately half the susceptibility of Gram-positive strains at equivalent AgNPs concentrations. Furthermore, smaller-diameter AgNPs demonstrate superior bactericidal efficacy, as their increased surface-area-to-volume ratio enhances interaction with microbial targets [86].

Particle concentration plays a crucial role in determining the antimicrobial efficacy of AgNPs, as demonstrated by multiple studies [83, 86]. Notably, smaller AgNPs (10 nm) exhibit superior antibacterial activity against non-biofilm-forming *Staphylococcus epidermidis*, achieving up to 76% bacterial reduction, whereas larger particles show reduced efficacy (58%). This size-dependent effect can be attributed to the higher surface-area-to-volume ratio of smaller nanoparticles, which enhances their interaction with bacterial membranes and facilitates greater silver ion (Ag⁺) release. In contrast, biofilm-forming *S. epidermidis* strains display heightened susceptibility to larger AgNPs (20–100 nm), with antimicrobial activity intensifying as particle size increases [25]. This phenomenon may stem from the ability of larger nanoparticles to physically disrupt the dense extracellular polymeric matrix of biofilms, allowing deeper penetration and sustained Ag⁺ release within the biofilm structure. Additionally, the prolonged retention of bigger AgNPs in the biofilm’s anionic microenvironment may enhance their bactericidal effects through cumulative oxidative stress and membrane damage. These findings underscore the importance of tailoring AgNP size and concentration based on bacterial strain characteristics, particularly their biofilm-forming capacity, to optimize antimicrobial performance [25, 83, 86].

Zinc oxide nanoparticles (ZnO-NPs) have emerged as promising antibacterial agents in biomedical applications, attributed to their cost-effectiveness, biocompatibility, sustained antimicrobial activity, and relatively low cytotoxicity [87]. In the present study, ZnO-NPs effectively inhibited *S. epidermidis* growth at a concentration of 250 µg/mL. This finding is consistent with previous research which has demonstrated the broad-spectrum antibacterial and biofilm-inhibitory properties of ZnO-NPs against various pathogens, including *S. epidermidis* [87]– [88].

The antimicrobial mechanisms of ZnO-NPs involve multiple pathways: (1) physical disruption of bacterial cell walls through direct nanoparticle interaction; (2) sustained release of Zn²⁺ ions, which interfere with cellular metabolism; (3) oxidation of thiol groups in glycolytic enzymes, impairing energy production; and (4) generation of reactive oxygen species (ROS), including superoxide anions (O₂⁻) and hydroxyl radicals, that induce oxidative damage to lipids, proteins, and DNA, ultimately leading to cell death [29]– [30, 87–89]. However, comparative analysis revealed that silver nanoparticles (AgNPs) exhibit superior anti-biofilm activity against strong biofilm-forming *S. epidermidis* strains [87]. This enhanced efficacy can be attributed to the smaller size of AgNPs, which increases their surface-area-to-volume ratio, facilitating deeper penetration into the biofilm matrix and more efficient interaction with bacterial cells [87, 90]. Additionally, Ag-NPs demonstrate stronger bactericidal effects due to their multifaceted mechanisms of action, including rapid silver ion release and potent ROS generation, making them particularly effective against biofilm-embedded bacteria [25, 27, 84]– [85].

To evaluate the relative potency of the silver nanoparticles (AgNPs), their antibacterial activity was benchmarked against vancomycin, a conventional therapeutic for *S. epidermidis*. Notably, the 10 nm AgNPs exhibited a minimum inhibitory concentration (MIC) of 1.25 µg/mL, representing a four-fold greater potency than vancomycin, which had an MIC of 4 µg/mL against the same strain. This enhanced efficacy can be attributed to the multi-faceted antibacterial mechanism of AgNPs. Specifically, they act through simultaneous pathways including membrane disruption, reactive oxygen species (ROS) generation, and biomolecular damage [25, 27, 83, 86]. Consequently, this complex mode of action presents a formidable challenge for bacteria, making the development of resistance via simple genetic mutations significantly more difficult compared to single-target antibiotics.

Moreover, AgNPs exert their effect by accumulating on the cell wall, where they form pores and pits that severely compromise its integrity and permeability. This physical damage impairs critical processes such as respiration, ultimately leading to apoptotic cell death [25, 85]. In direct contrast, vancomycin operates through a highly specific mechanism; as a glycopeptide antibiotic, it functions by inhibiting peptidoglycan polymerization, thereby preventing proper cell wall synthesis [91].

Another significant advantage of nanoparticles is their ability to penetrate bacterial biofilms, which are a major virulence determinant in *S. epidermidis* infections [58, 60]– [61]. This property offers a distinct clinical benefit over many antibiotics, which often demonstrate diminished efficacy within these protective structures [25, 83, 86]. While the development of resistance to antibiotics like vancomycin is a well-established clinical problem [12, 17], the non-specific, physical nature of nanoparticle interactions suggests a lower risk of resistance emergence [81]. This comparative analysis strongly positions the AgNP formulation as a promising alternative or adjunct agent to existing antibiotic regimens.

This study has potential limitations: The antimicrobial efficacy was evaluated against a single, albeit highly virulent, strain of *S. epidermidis*. While this provides a critical proof-of-concept against a specific emerging threat to tilapia, the generalizability of these findings may be limited. More significantly, the potent in vitro activity demonstrated here must be followed by in vivo challenge experiments in tilapia to verify therapeutic efficacy and ensure safety before clinical application can be considered.

While the phenotypic resistance profile is clearly defined, this study did not characterize the specific resistance genes (e.g., mecA, aacA-aphD, erm genes) or their genetic contexts (e.g., plasmids, genomic islands). Future work will prioritize elucidating the genetic determinants of resistance in *S. epidermidis* isolates through genetic mapping and whole-genome sequencing. Furthermore, we will explore the molecular interactions between the nanoparticles and these bacterial resistance mechanisms.

## Conclusion

This study establishes *Staphylococcus epidermidis* (MN153038) as an emerging pathogen responsible for summer mortality in tilapia. Notably, this strain demonstrates both high virulence and multidrug resistance, although it remains sensitive to vancomycin (MIC = 4 µg/mL). Virulence was found to be dose-dependent (3 × 10⁸–3 × 10⁹ CFU/mL) and also influenced by host factors; specifically, mortality rates ranged from 40% in adults to 95% in fingerlings. Furthermore, scale removal was shown to exacerbate mortality, increasing it by 40–60%. The observed pathology, particularly the deep, musculature-penetrating ulcers in fingerlings, underscores the significant threat this pathogen poses to intensive tilapia production.

The in-vitro antimicrobial efficacy of the nanoparticles revealed that 10-nm silver nanoparticles (AgNPs) demonstrated superior activity (MIC = 1.25 µg/mL) compared to their larger (100 nm) AgNPs and zinc oxide nanoparticles (ZnO-NPs; MIC = 125–250 µg/mL). The superior performance of the smaller AgNPs is likely a function of their enhanced biofilm penetration capabilities and higher surface-area-to-volume ratio, which augments their reactivity. Consequently, while nano-silver formulations present a promising therapeutic avenue, the limited potency of ZnO-NPs indicates a necessity for substantial formulation optimization.

## Supplementary Information

Supplementary Material 1.

## Data Availability

The datasets analysed during the current study are available in the GenBank database under the accession number: MN153038.
