# Supplementary material for: Emergence of multidrug-resistant Staphylococcus epidermidis in Nile tilapia (Oreochromis spp.): virulence, antimicrobial resistance, and nanoparticle-based control
Source: BMC Vet Res. 2026 Feb 2;22:124. doi: 10.1186/s12917-025-05213-w (PMC12930607; doi:10.1186/s12917-025-05213-w)
Supplement: Supplementary file 1 — Supplementary Material 1. [file 12917_2025_5213_MOESM1_ESM.docx]

**Supplementary data**

**Emergence of multidrug-resistant *Staphylococcus epidermidis* in Nile tilapia (*Oreochromis* spp.): Virulence, antimicrobial resistance, and nanoparticle-based control**

**Mahmoud Abou-Okada^1*^ and Engy Taha^1^**

^1^ Department of Aquatic Animal Medicine and Management, Faculty of Veterinary Medicine, Cairo University, Giza 11221 Egypt

*Corresponding author:

Mahmoud Abou-Okada

Department of Aquatic Animal Medicine and Management, Faculty of Veterinary Medicine, Cairo University, Giza 11221 Egypt

Email: [abouokada.mm@cu.edu.eg](mailto:abouokada.mm@cu.edu.eg)

**Supplementary data Pages: 5**

**Supplementary data Figures: 3**

**Methods**

**Phylogenetic analysis**

The 16S rRNA sequence acquired (MN153038) was then compared to existing databases through BLASTN on NCBI to determine their closest phylogenetic affiliations [1]. The neighbor joining algorithm in MEGA 12 was utilized to construct phylogenetic analysis [2]. The evolutionary history of the analyzed taxa was represented by constructing a bootstrap consensus tree, which was generated from 1000 replicates. The Maximum Composite Likelihood method, developed by Tamura *et al*.[3], was used to calculate evolutionary distances. These distances are expressed as the number of base substitutions per site [4].


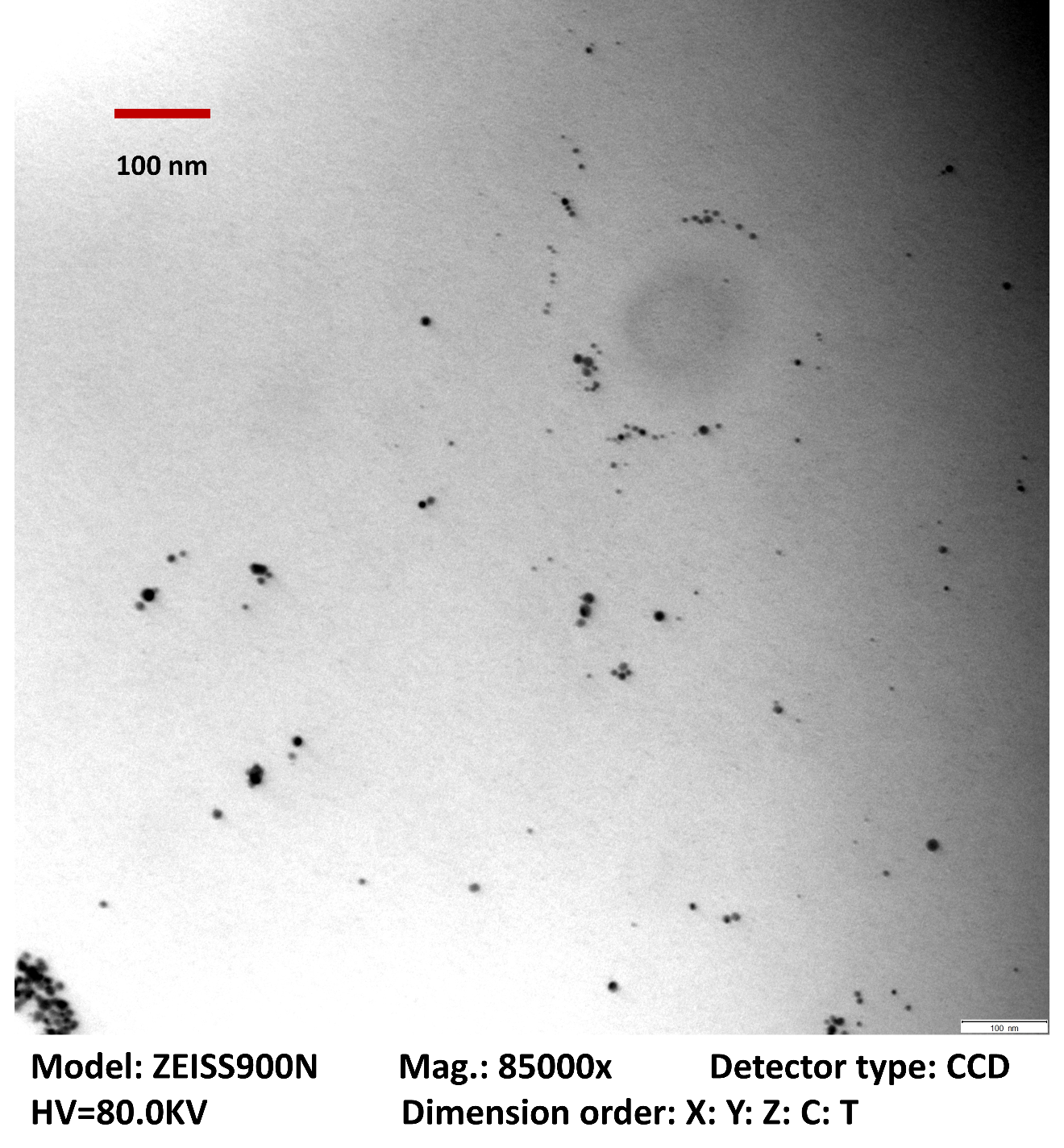


**Supplementary Figure 1. Transmission Electron Microscope image of silver nanoparticles (Ag NPs). The size of nanoparticles in the image are about 11.47 nm. The banner clearly displays TEM model (ZESIS900N), an accelerating voltage of 80.0 kV, and the magnification (85,000x). Scale bar represents 100 nm.**

**Results**


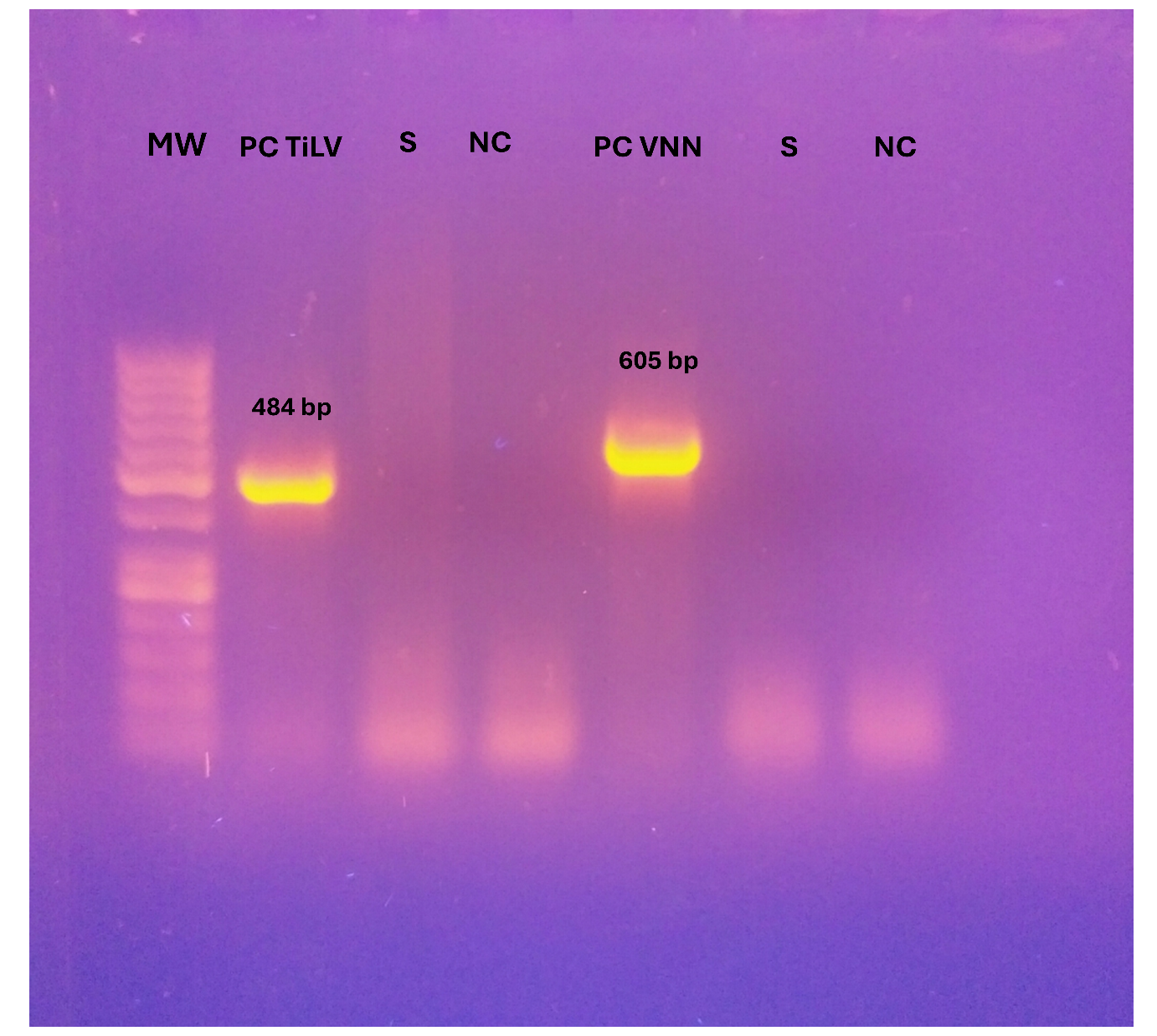


**Supplementary Figure 2.** Uncropped full-length labelled gel of TiLv and VNN. No detection of Tilapia lake virus (TiLV) RNA and virus nervous necrosis (VNN) RNA in tissues of diseased tilapia. MW: GeneRuler® 50 bp DNA Ladder (Thermo Fisher Scientific™), S: sample, NC: non-template negative control, PC: Positive control, TiLV: Tilapia lake virus and VNN: virus nervous necrosis.


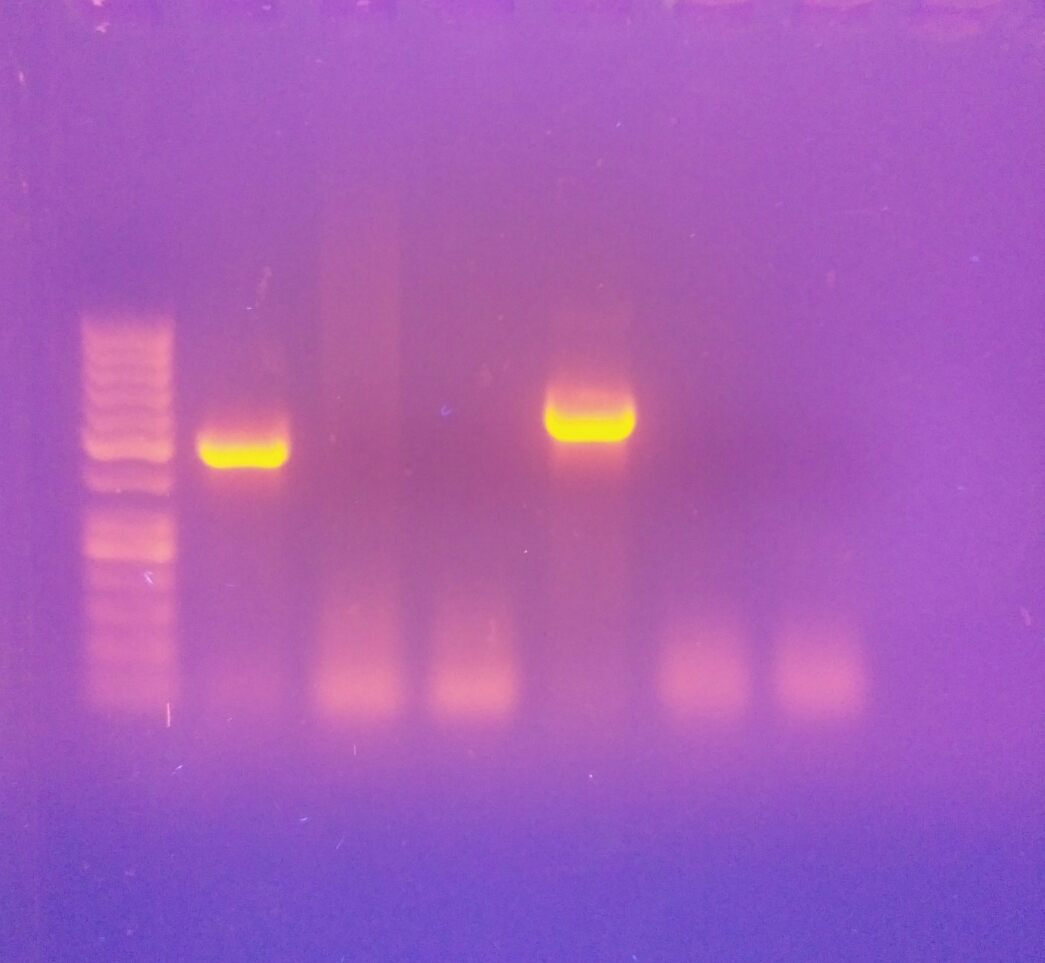


**Supplementary Figure 3.** Uncropped full-length unlabeled gel of TiLv and VNN.

**References**

[1] Altschul SF, Gish W, Miller W, Myers EW, Lipman DJ. Basic local alignment search tool. J. Mol. Biol. 1990;215(3):403–410. <https://doi.org/10.1016/S0022-2836(05)80360-2>

[2] Kumar S, Stecher G, Suleski M, Sanderford M, Sharma S, Tamura K. (2024). Molecular Evolutionary Genetics Analysis Version 12 for adaptive and green computing. Mol. Biol. Evol. 2024;41:1-9. <https://doi.org/10.1093/molbev/msae263>

[3] Tamura, K., Nei, M. & Kumar, S. Prospects for inferring very large phylogenies by using the neighbor-joining method. Proc. Nat. Acad. Sci. 2004 (USA) 101, 11030–11035. <https://doi.org/10.1073/pnas.0404206101>

[4] Felsenstein, J. Confidence limits on phylogenies: an approach using the bootstrap. Evolution. 1985;39:783–791. <https://doi.org/10.1111/j.1558-5646.1985.tb00420.x>
